# The redefinition of *Helicobacter pylori* lipopolysaccharide O-antigen and core-oligosaccharide domains

**DOI:** 10.1371/journal.ppat.1006280

**Published:** 2017-03-17

**Authors:** Hong Li, Tiandi Yang, Tingting Liao, Aleksandra W. Debowski, Hans-Olof Nilsson, Alma Fulurija, Stuart M. Haslam, Barbara Mulloy, Anne Dell, Keith A. Stubbs, Barry J. Marshall, Mohammed Benghezal

**Affiliations:** 1 West China Marshall Research Center for Infectious Diseases, Center of Infectious Diseases, Division of Infectious Diseases, State Key Laboratory of Biotherapy, West China Hospital, Sichuan University, Chengdu, China; 2 *Helicobacter pylori* Research Laboratory and Ondek Pty Ltd., School of Pathology & Laboratory Medicine, Marshall Centre for Infectious Disease Research and Training, University of Western Australia, Nedlands, Australia; 3 Department of Life Sciences, Imperial College London, South Kensington Campus, London, United Kingdom; 4 School of Chemistry and Biochemistry, University of Western Australia, Crawley, Australia; 5 Swiss Vitamin Institute, Route de la Corniche 1, Epalinges, Switzerland; Northwestern University, Feinberg School of Medicine, UNITED STATES

## Abstract

*Helicobacter pylori* lipopolysaccharide promotes chronic gastric colonisation through O-antigen host mimicry and resistance to mucosal antimicrobial peptides mediated primarily by modifications of the lipid A. The structural organisation of the core and O-antigen domains of *H*. *pylori* lipopolysaccharide remains unclear, as the O-antigen attachment site has still to be identified experimentally. Here, structural investigations of lipopolysaccharides purified from two wild-type strains and the O-antigen ligase mutant revealed that the *H*. *pylori* core-oligosaccharide domain is a short conserved hexasaccharide (Glc-Gal-DD-Hep-LD-Hep-LD-Hep-KDO) decorated with the O-antigen domain encompassing a conserved trisaccharide (-DD-Hep-Fuc-GlcNAc-) and variable glucan, heptan and Lewis antigens. Furthermore, the putative heptosyltransferase HP1284 was found to be required for the transfer of the third heptose residue to the core-oligosaccharide. Interestingly, mutation of HP1284 did not affect the ligation of the O-antigen and resulted in the attachment of the O-antigen onto an incomplete core-oligosaccharide missing the third heptose and the adjoining Glc-Gal residues. Mutants deficient in either HP1284 or O-antigen ligase displayed a moderate increase in susceptibility to polymyxin B but were unable to colonise the mouse gastric mucosa. Finally, mapping mutagenesis and colonisation data of previous studies onto the redefined organisation of *H*. *pylori* lipopolysaccharide revealed that only the conserved motifs were essential for colonisation. In conclusion, *H*. *pylori* lipopolysaccharide is missing the canonical inner and outer core organisation. Instead it displays a short core and a longer O-antigen encompassing residues previously assigned as the outer core domain. The redefinition of *H*. *pylori* lipopolysaccharide domains warrants future studies to dissect the role of each domain in host-pathogen interactions. Also enzymes involved in the assembly of the conserved core structure, such as HP1284, could be attractive targets for the design of new therapeutic agents for managing persistent *H*. *pylori* infection causing peptic ulcers and gastric cancer.

## Author summary

The bacterial pathogen *Helicobacter pylori* chronically infects the human stomach and causes ulcers and gastric cancer. The *H*. *pylori* lipopolysaccharide harbors unique properties that promote persistent infection through immune evasion. Despite the key role of *H*. *pylori* lipopolysaccharide in the bacterium’s pathogenesis, its precise domain organisation is still not available. Here, using a multidisciplinary approach involving biochemistry, genetics and analytical chemistry, we elucidated the *H*. *pylori* lipopolysaccharide structure and domain organisation. We found that the core domain is a short conserved hexasaccharide missing the canonical inner and outer core organisation. The O-antigen encompasses motifs previously assigned as the outer core domain, starting with a conserved trisaccharide, the variable glucan and heptan moieties and finishing, usually, with Lewis antigens. Furthermore, we demonstrate that the integrity of the core domain and the conserved trisaccharide of the O-antigen are critical for *H*. *pylori* to colonise the gastric niche. Together, the redefinition of the *H*. *pylori* lipopolysaccharide domains warrants future studies to dissect their roles in host-pathogen interactions and persistence. Also enzymes involved in the assembly of the conserved structure could be attractive targets for the design of new therapeutic agents for managing persistent *H*. *pylori* infection.

## Introduction

*Helicobacter pylori* is well-adapted to survival in the human stomach mucosa and establishes persistent infection, which causes chronic gastritis and can lead to peptic ulcer disease and gastric adenocarcinoma [1,2]. Lipopolysaccharide (LPS), a highly acylated glycolipid compactly anchored in the outer leaflet of the outer membrane (OM), is a key factor for *H*. *pylori* to establish colonisation and persistence in the gastric niche [3–7].

As a constituent biomolecule of most Gram-negative bacteria, the LPS is typically composed of three domains: the hydrophobic lipid A (or endotoxin), which anchors the molecule in the OM; the variable O-antigen extending from the cell to the external environment; and the core-oligosaccharide (which can be further divided into the inner and outer core), which links the O-antigen to the lipid A [8]. *H*. *pylori* constitutively modifies the *de novo* synthesized bi-phosphorylated and hexa-acylated KDO_2_-lipid A into a mono-phosphorylated and tetra-acylated KDO-lipid A to evade host immune surveillance and establish a persistent colonisation [9]. This unique lipid A structure confers *H*. *pylori* with the ability to resist cationic antimicrobial peptides (CAMPs), and to evade Toll-like receptor 4 (TLR-4) recognition [9]. In addition, the O-antigen of *H*. *pylori* LPS contains fucosylated oligosaccharides that mimic human Lewis antigens [5,10–12]. *H*. *pylori* is known to extensively vary its Lewis antigen expression pattern *in vivo*, which also contributes to its ability to evade host immune detection and adapt to the host environment during persistent infection [5].

**Fig 1:**
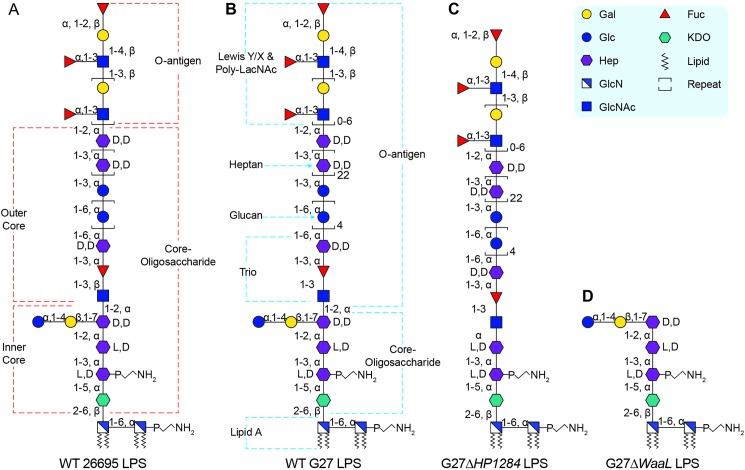
Previously proposed and the redefined LPS structure in *H*. *pylori*. The previously proposed LPS structure in strain 26695 wild-type (A), the redefined LPS structures of the G27 wild-type (B), G27Δ*HP1284* (C) and G27Δ*waaL* (D). The nomination of different domains of the LPS is annotated.

Our group has recently summarised the studied LPS structure and biosynthesis in *H*. *pylori* [13]. Being the first *H*. *pylori* strain with complete genome sequencing [14], the LPS structure of *H*. *pylori* strain 26695 is the most-studied and best-characterized [15–17]. The lipid A and Lewis antigens of *H*. *pylori* LPS have been well-characterised in terms of biosynthesis, structure and function [13]. However additional work is needed in regard to the characterization of the core-oligosaccharide domain. Similar to other Gram-negative bacteria [8], the core-oligosaccharide domain of 26695 is conceptually divided into two parts, the inner core and outer core [13]. The inner core is built as a conserved hexasaccharide (Glc-Gal-DD-Hep-LD-Hep-LD-Hep-KDO) and the first two LD-Hep residues (designated as Hep I and Hep II) are added sequentially by heptosyltransferases HP0279 and HP1191 [18]. However, the enzyme responsible for the transfer of the third DD-Hep residue (designated as Hep III) to Hep II remains to be identified. The outer core structure of *H*. *pylori* 26695 LPS was initially postulated to contain a DD-heptan with the first DD-Hep residue connecting a side-branched α-1,6-glucan [6,16,17,19–22], but a recent reinvestigation into the structure of 26695 LPS revised the outer core as being a linear arrangement of DD-heptan and α-1,6-glucan linked to the inner core through a trisaccharide (GlcNAc-Fuc-DD-Hep) termed as Trio [15] (**Fig 1A**). Furthermore, the attachment site of the O-antigen to the core-oligosaccharide has not been identified [13,15], and therefore the precise assignment of the O-antigen and core-oligosaccharide domains remains unclear.

Continuing the structural investigations of *H*. *pylori* LPS performed by the research groups of Trent [9,18,23–28], Moran [29–31], Altman [15,19,22,32–35] and Feldman [36,37], the goal of this study was to precisely define the core-oligosaccharide and O-antigen domains. The LPS of the *H*. *pylori* strain G27 was chosen to be analysed in this study, as this fully sequenced strain has been used extensively in *H*. *pylori* research [38], and for which the LPS structure has not been characterised. Using a combination of mass spectrometry and NMR spectroscopy, we redefined the core-oligosaccharide domain of *H*. *pylori* LPS to comprise solely the inner core conserved hexasaccharide of the previous model (Glc-Gal-DD-Hep-LD-Hep-LD-Hep-KDO, see annotations on **Fig 1B** and **Fig 1D**). Therefore, we propose that the *H*. *pylori* O-antigen domain includes the outer core structure of the previous model.

We further demonstrate that deletion of a conserved putative heptosyltransferase HP1284 from both G27 and X47 strains resulted in the attachment of O-antigen onto an incomplete core-oligosaccharide missing Hep III and the adjoining Glc-Gal unit (**Fig 1C**), suggesting that HP1284 is likely to be the Hep III transferase of the core-oligosaccharide domain. In addition, mutations of *HP1284* and *waaL* led to increased sensitivity to polymyxin B and loss of colonisation in the mouse model compared to wild-type. Mapping the mutagenesis and colonisation data of previous studies onto the newly defined *H*. *pylori* LPS core-oligosaccharide and O-antigen domains suggests that the conserved Trio and the intact core domains are critical for colonisation.

## Results

### The core-oligosaccharide and O-antigen domains of *H*. *pylori* G27 LPS are similar to that of strain 26695

Using preparative isolation, highly pure LPS from wild-type G27 was obtained for MS structural analysis. GC-EI-MS analysis of monosaccharides as their TMS (trimethylsilyl) derivatives (**Fig A in S1 Text**) revealed that wild-type G27 LPS contains Fuc, Gal, Glc, Hep, GlcNAc and KDO. Methylation linkage analysis (**Table A in S1 Text**) indicated a complex monosaccharide composition including terminal and 3-linked Fuc; terminal, 2-, 3- and 4-linked Gal; terminal, 3-, and 6-linked Glc; 2- and 3-linked DD-Hep; 2-, 3-, 7- and 2,7-linked Hep; and terminal, 3-, 4- and 3,4-linked GlcNAc. Overall the monosaccharide composition of wild-type G27 LPS is very similar to the recently re-investigated LPS from strain 26695 [15]. Terminal and 2-linked Gal, and 3,4-linked GlcNAc that are characteristics of the LacNAc element of Lewis antigens were found, together with terminal Fuc, suggesting the existence of both Le^x^ and Le^y^ epitopes that are observed in the LPS of *H*. *pylori* strains including 26695 [6,7,15–17], SS1 [6,16,32], J99 [6], NCTC11637 [16].

**Fig 2:**
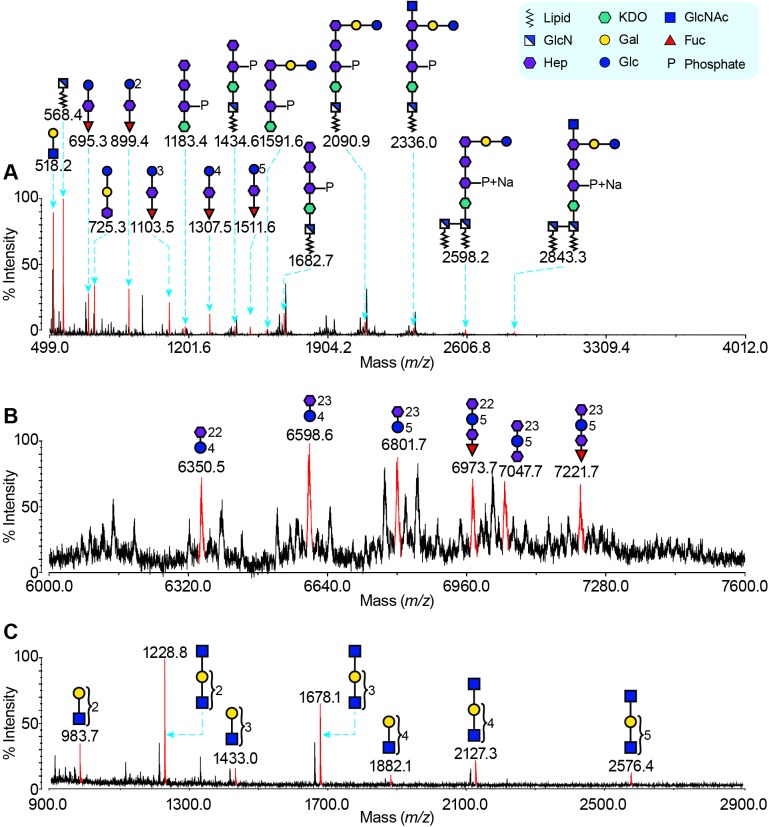
MS analysis of *H*. *pylori* Wild-type G27 LPS. Wild-type G27 LPS samples were subjected to (A): methanolysis; (B): mild HF hydrolysis, and (C): mild periodate oxidation, respectively. MALDI-TOF MS spectra were recorded after permethylation. The MS peaks corresponding to sodiated glycans are coloured red, and annotated with *m/z* values and glycan structures. Note that for the spectrum after mild HF hydrolysis, the most intense isotopic peaks are annotated. Other blank signals are mainly due to (A): an addition of a sodium atom; and (C): incomplete reduction. The MS data indicate the fundamental architecture of wild-type G27 LPS is the same as strain 26695, containing LacNAc, heptan, glucan, Trio, the phosphorylated Glc-Gal-tri-Hep-KDO structure and lipid A.

The wild-type G27 LPS was subjected to methanolysis to facilitate further MS analysis as intact LPS molecules are normally too large for direct MS characterisation. Matrix-assisted laser desorption/ionization time of flight (MALDI-TOF) mass fingerprints of the methanolysis products of the wild-type G27 LPS sample after permethylation are shown in **Fig 2A**. The annotation of MS peaks was based on the previously characterised strain 26695 LPS and monosaccharide composition provided by the GC-EI-MS analyses. An MS peak at mass-to-charge ratio (*m/z)* 518.2 shown in **Fig 2A** was annotated as LacNAc, and a peak at *m/z* 568.4 was annotated as *N*-acylated-glucosamine from lipid A. A cluster of MS peaks at *m/z* 695.3, 899.4, 1103.5, 1307.5 and 1511.6 shown in **Fig 2A** was assigned to glucan-Hep-Fuc structures with Glc repeating from one to five times respectively. A peak at *m/z* 2598.2 in **Fig 2A** corresponds to a phosphorylated Glc-Gal-tri-Hep-KDO-lipid A structure whose methanolysed products give rise to most other peaks in the spectrum. The Trio is cleaved into two parts: the GlcNAc remains attached to the phosphorylated Glc-Gal-tri-Hep-KDO structure, giving rise to peaks at *m/z* 2843.3 and 2336.0 in **Fig 2A**, and the -DD-Hep-Fuc portion is attached to the previously mentioned glucan clusters. Since all the components of the wild-type G27 LPS are also found in the LPS of strain 26695, we propose that both LPS molecules are structurally very similar (compare **Fig 1A** and **Fig 1B**).

To characterise the heptan in the wild-type G27 LPS, mild HF hydrolysis was used to cleave the 1,3-linked Fuc-GlcNAc glycosidic bond in the Trio moiety so that the entire heptan-glucan structure could be observed. The MALDI-TOF spectrum of wild-type G27 LPS after mild HF hydrolysis and permethylation gives an MS pattern of glycan fragments strongly suggesting that the heptan and glucan can be as long as 23 Hep and 5 Glc units respectively (**Fig 2B**). This full-length heptan-glucan structure gives rise to a peak at *m/z* 7721.7.

To further determine the length of the poly-LacNAc portion, wild-type LPS was mildly oxidised using sodium periodate and the MALDI-TOF spectrum was recorded (**Fig 2C)**. Periodate oxidation cleaves glycans between neighbouring hydroxyl groups, therefore poly-LacNAc/Lewis structures survive the reaction while most other glycan structures are completely oxidised. Notably, for the Le^y^ epitope, two terminal Fuc residues were oxidised, leaving intact LacNAc structures; whereas for the Le^x^ epitope, terminal Gal and Fuc were oxidised, leaving GlcNAc-LacNAc structures. As expected, two clusters of peaks at *m/z* 1228.8, 1678.1, 2127.3 and 2576.4, and at *m/z* 983.7, 1433.0 and 1882.1 that are representative of the Le^x^ and Le^y^ epitopes respectively can be observed in the MS spectrum (**Fig 2C**). The longest observed poly-LacNAc has 6 repeating units.

Collectively, our data suggest that the fundamental architecture of G27 wild-type LPS is very similar to strain 26695. They both contain fucosylated LacNAc (Le^x^ and Le^y^) (**Fig B in S1 Text**), heptan, glucan, Trio, a phosphorylated Glc-Gal-tri-Hep-KDO structure and lipid A.

### *H*. *pylori* G27 *HP1284* encodes putative Hep III transferase of the core LPS

**Fig 3:**
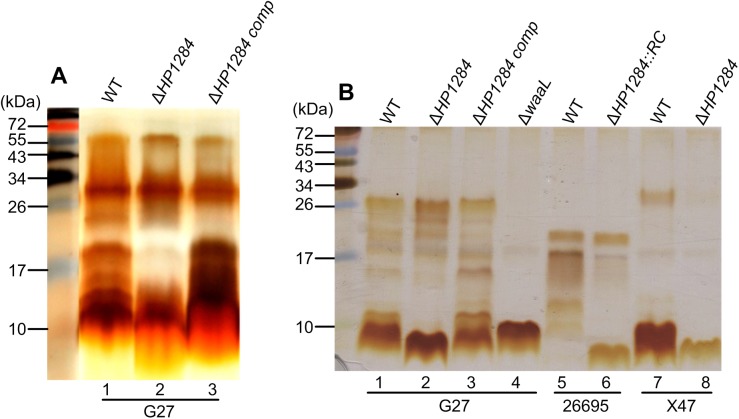
Effects of *HP1284* and *waaL* mutation on *H*. *pylori* LPS. LPS samples from *H*. *pylori* wild-type and mutants were analysed by SDS-PAGE and silver stain. (A): Low resolution SDS-PAGE. Lane 1–3: G27 wild-type, *HP1284* deletion and *HP1284* complementation in strain G27; (B): High resolution Tricine-SDS-PAGE. Lane 1–4: wild-type, *HP1284* deletion, *HP1284* complementation and *waaL* deletion in strain G27; Lane 5–6: wild-type and *HP1284* insertion mutant in strain 26695; Lane 7–8: wild-type and *HP1284* deletion mutant in strain X47.

Not all glycosyltransferases responsible for *H*. *pylori* LPS assembly have been identified [13]. Of particular interest is the heptosyltransferase responsible for the transfer of Hep III to which the conserved Trio motif is attached (**Fig 1A**). A targeted approach to identify the Hep III transferase of the core LPS was based on the identification of highly conserved putative glycosyltranferases in the *H*. *pylori* genome [13]. The putative protein sequence of HP1284 in strain G27 was found to share 36% identity to LPS Hep III transferase WaaQ from *Haemophilus influenza* 86-028NP. Therefore, the corresponding mutant G27Δ*HP1284* was constructed. Silver staining of LPS extracted from G27Δ*HP1284* displayed a similar pattern to the wild-type LPS, although it appeared to be missing bands sized around 15–20 kDa LPS (**Fig 3A, lane 2**), indicating that HP1284 is involved in the biosynthesis of LPS. Genetic complementation of G27Δ*HP1284* mutant restored the wild-type LPS profile (**Fig 3A, lane 3**). To gain higher resolution of the core region, Tricine-SDS-PAGE was used to separate the low molecular weight LPS species more effectively. This revealed that mutation of *HP1284* in G27 resulted in a clear change in bands of low molecular weight LPS (about 10 kDa) corresponding to core lipid-A (**Fig 3B, lane 2**). The same profile was also observed for *HP1284* isogenic mutants made in strains 26695 and X47 (**Fig 3B, lanes 6** and **8**) demonstrating that this gene’s role in the biosynthesis of the *H*. *pylori* LPS core region is conserved across different strains. Upon genetic complementation of G27Δ*HP1284* mutant, the 10 kDa band species on Tricine-SDS-PAGE were restored (**Fig 3B, lane 3**).

**Fig 4:**
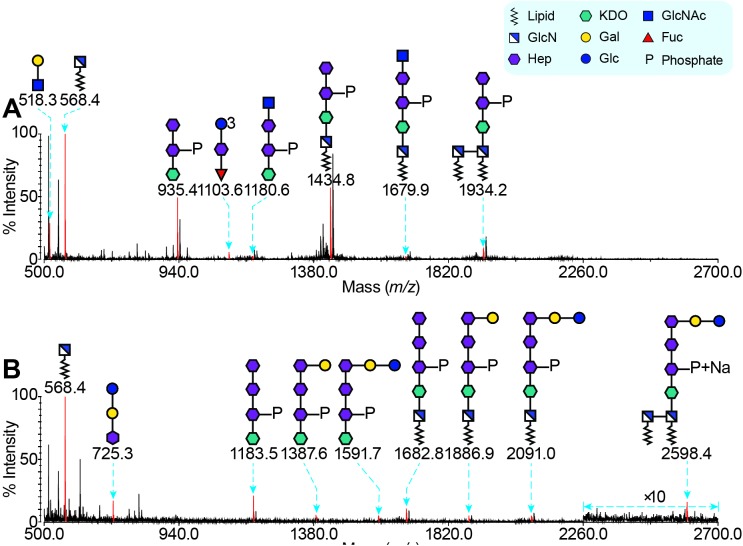
MS Analysis of LPS from G27 *HP1284* and *waaL* Deletion Mutants. The LPS samples were methanolysed, permethylated and analysed by MS. MALDI-TOF spectra of LPS from G27 *HP1284* and *waaL* deletion mutants are shown in (A) and (B), respectively. The MS peaks corresponding to sodiated heptan-glucan structures are coloured red and annotated with *m/z* values. Most blank peaks are due to contamination and the addition of a sodium atom. The MS data indicate the core-oligosaccharide of G27 LPS is a hexa-saccharide with a sequence of Glc-Gal-Hep-Hep-Hep-KDO. The deletion of *HP1284* leads to an incompletely synthesized core, which does not affect its O-antigen.

To gain further insight into the role of *HP1284* gene in the *H*. *pylori* LPS biosynthesis, the same MS-based strategies were used for analysing the structure of LPS purified from G27Δ*HP1284*. The MS spectra of the mild HF hydrolysed and sodium periodate oxidised LPS from G27Δ*HP1284* were very similar to the wild-type strain (**Fig C in S1 Text**), indicating the existence of poly-LacNAc, heptan, glucan and the Trio moiety. However, the MS spectrum of permethylated G27Δ*HP1284* LPS after methanolysis presented a distinct MS pattern (**Fig 4A**). The MS peak at *m/z* 1183.4 in **Fig 2A** corresponding to the phosphorylated GlcNAc-tri-Hep-KDO structure shifted to a peak at *m/z* 1180.6 in **Fig 4A**. Analysis using MALDI-TOF/TOF of this peak suggested that it corresponded to a phosphorylated glycan with a sequence of GlcNAc-Hep-Hep-KDO (**Fig C in S1 Text**), which indicated that the Hep III residue of the core together with the Glc-Gal disaccharide attached to it were missing. Methylation linkage analysis also provided supportive data, *i*.*e*., no terminal-Glc, 4- linked Gal or 2,7-linked Hep was found (**Table A in S1 Text**).

Together, the sequence homology of HP1284 to the LPS Hep III transferase WaaQ of *H*. *influenza* and the genetic and structural data presented above indicate that the conserved putative heptosyltransferase HP1284 is very likely to be the Hep III transferase, participating in the biosynthesis of the core-oligosaccharide of *H*. *pylori* LPS.

### *H*. *pylori* LPS core-oligosaccharide domain is short and missing the canonical outer and inner core organisation

The *HP1284* mutation in the core-oligosaccharide did not affect further LPS synthesis and the function of O-antigen ligase WaaL, though it did lead to a simultaneous loss of the Hep III residue of the core and the Glc-Gal disaccharide. To precisely identify the O-antigen attachment site, and to define the core and O-antigen domains of *H*. *pylori* LPS, we analysed the LPS structure of the *waaL* mutant that lacks the O-antigen and only harbours core-lipid A [36]. Therefore, core-lipid A was purified from the *waaL* deletion mutant G27Δ*waaL* (**Fig 3B, lane 4**) to carry out methanolysis/MS analysis. No peak corresponding to the LacNAc, heptan, glucan and Trio was observed, suggesting that the core-oligosaccharide in *H*. *pylori* G27Δ*waaL* lacks all the glycan structures from the Trio outwards (**Fig 4B**).

An MS peak at *m/z* 1591.7 corresponding to a short core-oligosaccharide with a sequence of Glc-Gal-tri-Hep-KDO was observed (**Fig 4B**). MS peaks at *m/z* 1886.9 and 2598.4 that are indicative of core-lipid A structures were also observed (**Fig 4B**). GC-EI-MS linkage analysis revealed a much simpler monosaccharide composition including terminal-Glc, 4-linked Gal, 2-, 3- and 7-linked Hep (**Table A in S1 Text**).

To further confirm the structure of G27Δ*waaL* LPS, NMR experiments on intact LPS were carried out for LPS samples in D_2_O containing deuterated dodecylphosphocholine (D_38_-DPC), thus anchoring the lipid tails into micelles. This technique has been used previously for the study of glycolipids [39] and rough-type LPS [40]. Tentative assignments of some resonances in the ^1^H and ^13^C spectra of the G27Δ*waaL* LPS derived from analysis of 2D TOCSY and NOESY spectra are listed in **Table B in S1 Text**. Seven anomeric proton signals attributable to three α-Hep, one α-GlcN, one α-Glc, one β-GlcN and β-Gal were observed, which is fully consistent with our MS data and previous research [32]. In accord with our MS experiments, the NMR data suggest that the G27Δ*waaL* LPS is truncated from the Trio, indicating that *H*. *pylori* G27 synthesises a very short core-oligosaccharide with a sequence of αGlc1-4βGal1-7αHep1-2αHep1-3αHep-KDO.

Deletion of the *waaL* gene in *H*. *pylori* strain X47 also resulted in a short core LPS (**Fig D in S1 Text**). In addition, double mutation of *HP1284* and *waaL* led to further reduction in the size of the core-oligosaccharide, supporting that *HP1284* encodes for the putative heptosyltransferase that is responsible for transfer of the Hep III residue (**Fig D in S1 Text**). The LPS molecules purified from X47 wild-type and X47Δ*HP1284* were subjected to methanolysis and analysed by MS. The resulting data indicate that X47 LPS shares the same structural architecture with G27 and 26695 LPS (**Fig E in S1 Text**). In addition, the deletion of *HP1284* in the X47 background led to the same structural change with the core-oligosaccharide missing the Hep III residue, as seen in G27Δ*HP1284* (**Fig E in S1 Text**).

Together, these results led to the redefinition of the core and O-antigen domains of *H*. *pylori* LPS and to the identification of the putative Hep III transferase of the core-oligosaccharide domain (**Fig 1B, 1C and 1D**).

### Deletion of *HP1284* or *waaL* leads to a moderate increase in polymyxin B susceptibility

Taking into consideration the redefinition of the *H*. *pylori* LPS core-oligosaccharide and the O-antigen domains in this study, we assessed the roles played by the putative Hep III transferase HP1284 and the O-antigen ligase WaaL in *H*. *pylori*'s resistance to CAMPs. Polymyxin B has a similar mechanism of action to CAMPs, and therefore is an experimental substitute for CAMPs in laboratory settings [9,18]. Minimal inhibitory concentration (MIC) of polymyxin B was determined for the *HP1284* and *waaL* mutants in three different *H*. *pylori* strains G27, X47 and 26695, using polymyxin B Etest strips. As the KDO hydrolase (HP0579/HP0580) mutation confers strong sensitivity to polymyxin B due to a deficiency in lipid A modification [18], it was introduced in strain G27 and X47 (G27Δ*HP0579* and X47Δ*HP0579*-*HP0580* mutants respectively*)* for comparative determination of polymyxin B MICs.

Resistance to polymyxin B varied substantially among three *H*. *pylori* wild-type strains, with MICs of 4.8 ± 1.1, 332.8 ± 70.1 and 149.3 ± 37.0 μg/mL in G27, X47 and 26695, respectively (**Table 1**). As expected, KDO hydrolase mutants G27Δ*HP0579* and X47Δ*HP0579*-*HP0580* showed a marked 37.0 and 1147.0 fold decrease in resistance to polymyxin B when compared to their corresponding wild-type strains. The *HP1284* mutant in G27, X47 and 26695 showed a 5.3, 3.1 and 2.5 fold decrease in resistance to polymyxin B, and the *waaL* mutant in G27 and X47 showed a 3.7 and 5.7 fold decrease in resistance to polymyxin B.

**Table 1:** Polymyxin B Minimal Inhibitory Concentration (MIC) of *H*. *pylori* G27, X47 and 26695 Wild-type Strains and LPS Mutants.

Strains	Role in LPS biosynthesis	Polymyxin B MIC (μg/mL)
G27 wild-type	Full-length LPS	4.8 ± 1.1
G27Δ*HP1284*	Putative Hep III transferase	0.9 ± 0.1
G27Δ*waaL*	O-antigen ligase	1.3 ± 0.3
G27Δ*HP0579*	KDO hydrolase	0.13 ± 0.0
X47 wild-type	Full-length LPS	332.8 ± 70.1
X47Δ*HP1284*	Putative Hep III transferase	106.7 ± 16.5
X47Δ*waaL*	O-antigen ligase	58.7 ± 9.2
X47Δ*HP0579*-*HP0580*	KDO hydrolase	0.23 ± 0.03
26695 wild-type	Full-length LPS	149.3 ± 37.0
26695 *HP1284*::*RC*	Putative Hep III transferase	58.7 ± 9.2

MIC are reported as μg/mL and are the average of three experiments using polymyxin B Etest strips (Biomerieux) on CBA plates.

Compared to the severe decrease in polymyxin B resistance of the KDO hydrolase mutant, the *HP1284* and *waaL* mutants only exhibited a moderate decrease in resistance to polymyxin B.

### Deletion of *HP1284* and *waaL* leads to loss of colonisation in the mouse model

We investigated the role of HP1284 in colonisation of the gastric mucosa using strain X47, a robust mouse coloniser [41]. Two independent sets of mouse experiments were performed using C57BL/6J mice. In both Experiment 1 and 2, two weeks after oral challenge, the X47 wild-type strain could establish colonisation within the mouse stomach, whereas no bacteria could be recovered from the mice challenged with X47Δ*HP1284* (**Table 2**). This suggests that HP1284 is required by *H*. *pylori* strain X47 for host colonisation.

**Table 2:** Viable Counts of *H*. *pylori* X47Δ*HP1284* and X47Δ*waaL* mutants Recovered from Mice at 2 or 8 Weeks post Challenge*.

Inoculum strain	Log_10_ CFU (mean ± SD)	
	Week 2	Week 8
**Experiment 1** (n = 5)		
X47	6.24 ± 0.22	
X47**Δ***HP1284*	BDL**	
**Experiment 2** (n = 10)		
X47	4.95 ± 0.55	
X47**Δ***HP1284*	BDL	
**Experiment 3** (n = 5)		
X47	6.31 ± 0.24	5.87 ± 0.69
X47**Δ***waaL*	BDL	BDL

* The data presented are the mean log_10_ CFU ± SD of n = 5 or 10 mice per group.

**BDL, below detectable limit <1.7 log_10_ CFU).

To assess the mutation of WaaL on colonisation, a third mouse experiment was performed. Again, X47 wild-type strain colonised the mice well by weeks 2 and 8. However, X47Δ*waaL* strain was unable to colonise C57BL/6J mice at the two time points (**Table 2**), suggesting that the O-antigen ligase WaaL is also required for the survival of *H*. *pylori* strain X47 within a host.

## Discussion

In this study, a combination of genetic and structural analysis of *H*. *pylori* LPS from wild-type and mutant strains enabled the experimental identification of the O-antigen attachment site and the precise assignment of the core-oligosaccharide and O-antigen domains. In addition, HP1284 is proposed to encode the Hep III transferase of the LPS core domain, based on structural analysis of corresponding mutant LPS in two strains, sequence homology to the Hep III transferase WaaQ of *H*. *influenza*, and the reduction in the size of the core-oligosaccharide of the double *HP1284*/*waaL* mutant compared to the single *waaL* mutant. Deletion of *HP1284* and the O-antigen ligase *waaL* led to a moderate decrease in resistance to polymyxin B and loss of colonisation in the mouse model.

**Fig 5:**
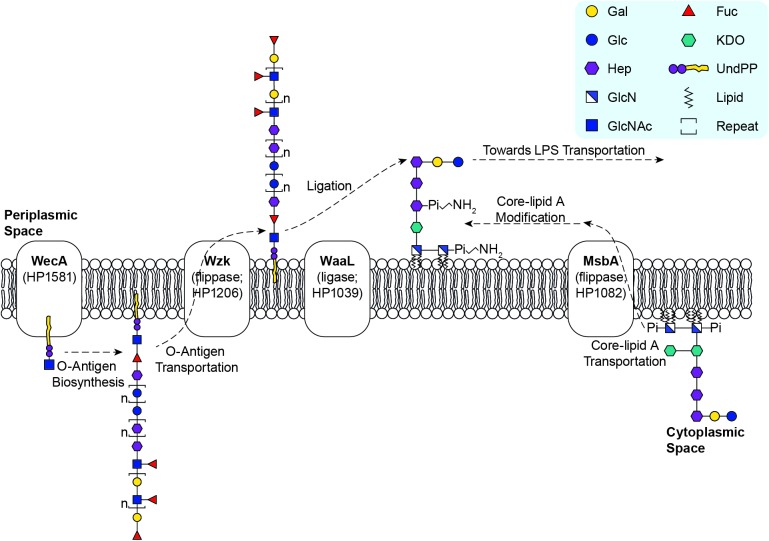
Proposed Model for the LPS Biosynthetic Pathways in *H*. *pylori*. *H. pylori* LPS biosynthesis follows a novel Wzk-dependent pathway [36]. The assembly of the very long O-antigen occurs in the cytoplasm and is initiated by WecA [36] transferring the GlcNAc residue of the Trio onto the UndPP carrier. Successive glycosyltransferases are recruited to complete the synthesis of the whole O-antigen encompassing the conserved Trio and variable glucan, heptan and Lewis antigens. After assembly in the cytoplasm, the UndPP-linked O-antigen is translocated by flippase Wzk to the periplasm [36]. Also assembled in the cytoplasm, the de novo synthesized core-lipid A is flipped by flippase MsbA to the periplasm [28]. After constitutive modifications through dephosphorylation and deacylation [9], the modified core-lipid A is ligated with O-antigen by the ligase WaaL [36], and the Hep III residue is the attachment site.

The structural analysis of core-oligosaccharide accumulating in the O-antigen ligase mutant, G27Δ*waaL*, enabled the identification of the Hep III residue as the precise attachment site of the *H*. *pylori* O-antigen (**Fig 5**). The core-oligosaccharide in the G27Δ*waaL* mutant is a short hexa-saccharide comprised of Glc-Gal-DD-Hep-LD-Hep-LD-Hep-KDO (**Fig 1D**), indicating that the missing glycan structure, from the Trio outwards, is transferred as the O-antigen by ligase WaaL to the Hep III residue. As O-antigen biosynthesis in *H*. *pylori* is initiated in the cytoplasm through the action of WecA transferring a GlcNAc onto a undecaprenyl phospholipid (UndPP) carrier [36] (**Fig 5**), we propose that the first GlcNAc residue in the Trio, not the GlcNAc of the Lewis antigen, is the first sugar of the long *H*. *pylori* LPS O-antigen encompassing the Trio, the glucan, the heptan and Lewis antigens (**Fig 1B**). This finding challenges the previous LPS model with an inner core and an outer core decorated with an O-antigen composed of Lewis antigens only [16] (**Fig 1A**).

The linear architecture of the heptan-glucan-Trio of the G27 LPS structure is consistent with the recently revised structures of LPS in strains 26695 [15], SS1 [32] and serogroup O:3 [34], supporting that the linearity of this region may be a general feature of *H*. *pylori* strains. This overturns the paradigm of *H*. *pylori* LPS outer core containing a DD-heptan with the first DD-Hep residue connecting a side-branched glucan in earlier studies [6,16,17,19–22]. Furthermore, our study demonstrated the presence of the Trio in both G27 and X47 LPS structures. This moiety has also been reported in the revised LPS structures of 26695 [15], SS1 [32] and serogroup O:3 [34], suggesting that the Trio is a common LPS feature of *H*. *pylori* strains. Conservation of the Trio among *H*. *pylori* strains contrasts with the variability of the distal domains of O-antigen such as glucan, heptan and Lewis antigen in 26695 [15], and a short oligomer of 1,2-linked ribofuranose (riban) and Lewis antigen in SS1 [32].

Of note, the O-antigen in *HP1284* deletion mutant is transferred onto an incomplete core-oligosaccharide missing the Hep III and attached disaccharide (**Fig 1C**). This suggests that when the Hep III residue is missing, *H*. *pylori* WaaL can still use the Hep II residue as the attachment site for the O-antigen ligation, and that the branched disaccharide in the core-oligosaccharide is not relevant for the activity of *H*. *pylori* WaaL. This differs from *Escherichia coli* and *Salmonella enterica* in which the terminal GlcNAc residue in the core-oligosaccharide acceptor is crucial for recognition by WaaL [42,43]. However, it has been shown that a *waaL* mutant of *E*. *coli* cannot be cross-complemented by the *waaL* gene of *H*. *pylori* [36], supporting that WaaL in different bacteria has high specificity for its cognate core-oligosaccharide [37,44].

**Fig 6:**
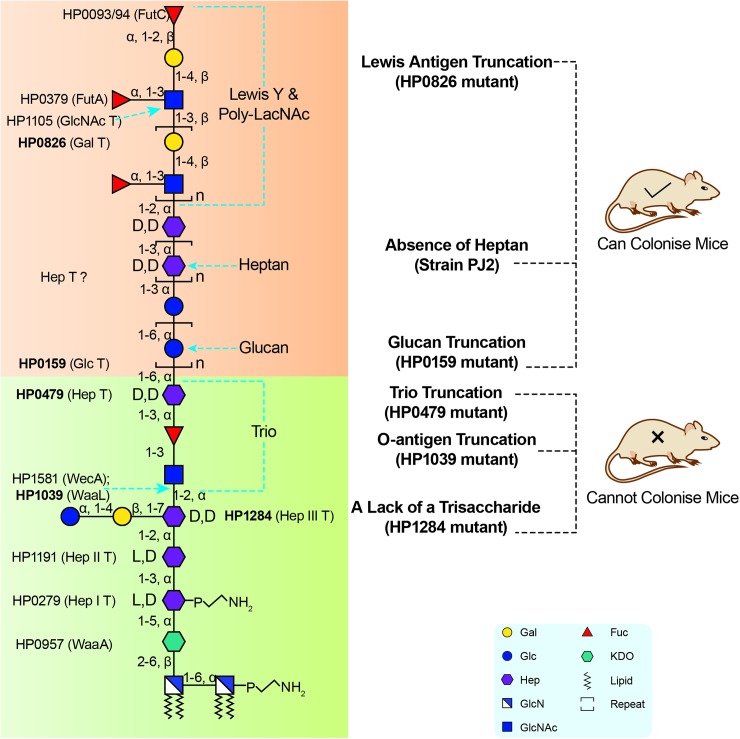
The conserved Trio and the short core of *H*. *pylori* LPS are important for colonisation. Currently known glycosyltransferases are assigned to the redefined complete structure of *H*. *pylori* LPS. The lipid A, the core-oligosaccharide domain and the Trio are proposed to be conserved and are important for host colonisation, whereas the Lewis antigen, heptan and glucan are variable and non-essential for colonisation.

Prior to this study, glycosyltransferases responsible for transferring the Hep III, the GlcNAc and Fuc residues of the Trio, and the heptan were unknown [13]. Here the targeted discovery of the highly conserved putative heptosyltransferase gene *HP1284* combined with our genetic and structural data support that HP1284 is the Hep III transferase (**Fig 1C**). As discussed earlier, WecA can now be considered the enzyme transferring the GlcNAc residue of the Trio, whereas the fucosyltransferase of Trio is yet to be identified. Combined with the previously characterised LPS biosynthetic genes, currently known glycosyltransferases were assigned to the complete structure of *H*. *pylori* G27 LPS (**Fig 6**).

To assess the role of the core-oligosaccharide and O-antigen domains in CAMPs resistance, polymyxin B MICs were measured in corresponding mutants and compared to the KDO hydrolase mutant. This mutant is highly sensitive to CAMPs due to deficiency in the constitutive modifications of lipid A [18], the primary mechanism involved in *H*. *pylori*'s resistance to CAMPs [9]. In this study, the KDO hydrolase mutants displayed a 37- and 1147-fold higher susceptibility, in G27 and X47 respectively, confirming the key role of lipid A modification in resistance to CAMPs in *H*. *pylori*. In contrast, the lack of O-antigen in the *waaL* mutant or the absence of core Hep III and adjoining Glc-Gal disaccharide in the *HP1284* mutant only led to a 3- to 5-fold increase in susceptibility to polymyxin B, suggesting that *HP1284* or *waaL* mutations is unlikely to affect the highly-ordered lipid A modification process. Of note, polymyxin B MICs varied almost 70-fold among the three tested wild-type *H*. *pylori* strains with MICs increasing in the following order, G27, 26695 and X47. This suggests that strain specific mechanisms other than the constitutive lipid A modifications also contribute to resistance to CAMPs. For example, the incorporation of host cholesterol onto the surface of *H*. *pylori* [45], or the regulation of the glycerophospholipid content in the OM as reported in *S*. *typhimurium* [46] may also be critical for CAMP resistance.

Finally, mapping LPS mutagenesis and colonisation data of previous studies onto the redefined *H*. *pylori* LPS structure suggested that the conserved Trio and the short core of *H*. *pylori* LPS are important for colonisation whereas the less conserved domains are less important (**Fig 6**). For example, the *HP0826* mutant in SS1 strain, which is devoid of Lewis antigen, is still able to colonise the host, although less efficiently compared to the wild-type [16,35]. The DD-heptan and the glucan of the O-antigen are not essential for colonisation [19,33,47], while the *HP0479* mutant truncated at the Hep residue of the Trio resulted in loss of colonisation ability [22]. The variability of the O-antigen beyond the conserved Trio may enable *H*. *pylori* to adapt to a specific host for example via host mimicry and the presence of Lewis antigens. In contrast, the conserved core and Trio may reflect evolutionary adaptation to shared mammalian traits such as the innate immune system, enabling the survival and persistence of *H*. *pylori* within the gastric mucosa.

The requirement of a conserved LPS moiety for gastric colonisation was also observed in this study. The loss of mouse colonisation in the X47**Δ***waaL* mutant could be attributed to the truncation of the conserved Trio moiety rather than the remaining variable regions. However, the inability of the X47**Δ***HP1284* mutant to colonise the mouse stomach is intriguing as the LPS structure in this mutant lacks only three carbohydrate units of the core-oligosaccharide yet still harbours the extended O-antigen. In addition, the mutant exhibited only a moderate susceptibility to polymyxin B. This warrants further investigation into the role of the structure of the core-oligosaccharide in host colonisation.

In summary, we propose the redefinition of the core-oligosaccharide and O-antigen domains of *H*. *pylori* LPS. HP1284 is found to be the putative Hep III transferase, and its mutation leads to an abnormal core-oligosaccharide but does not affect the O-antigen ligation. Future studies are needed to elucidate the essential role of HP1284 in host colonisation.

## Materials and methods

### Bacterial strains and culture conditions

The bacterial strains and plasmids and oligonucleotides used in this study are listed in **Table C** and **D** respectively **in S1 Text**. *H*. *pylori* strains were cultured on Columbia Blood Agar (CBA) plates, supplemented with 5% horse blood and 5% new-born calf serum (NCS). Antibiotic selection in *H*. *pylori* was carried out by supplementing media with chloramphenicol (10 μg/mL) or streptomycin (10 μg/mL) where appropriate. Plates were incubated at 37°C for 24∼48 h under microaerobic conditions established in sealed jars using the Anoxomat MarkII system (Mart Microbiology B.V., the Netherlands) after one atmosphere replacement with the following gas composition N_2_:H_2_:CO_2_, 85:5:10.

### Construction of *H*. *pylori* LPS mutants

#### The deletion of *waaL* and *HP0579* in strain G27

G27Δ*waaL* and G27Δ*HP0579* were generated using a highly efficient Xer-cise gene deletion method [48]. Following the four step methodology, mutants were made for *waaL* using primer set WaaL, and for *HP0579* using primer set HP0579 (**Table C in S1 Text**). The resulting plasmids pWaaL-AB-difH-RC and p0579-AB-difH-RC were used to transform strain G27 to generate G27Δ*waaL* and G27Δ*HP0579*, respectively.

#### The mutation of *HP1284* in strains 26695 and G27

The G27 *HP1284* insertion mutant was generated using a MuA transposase methodology previously described [49]. Primers HP1284F and HP1284R were used to produce plasmid pCR2.1-1284-RC, which was used to transform strain G27 to generate G27*HP1284*::*RC*. The genomic DNA from G27*HP1284*::*RC* was used to transform strain 26695 to generate mutant 26695*HP1284*::*RC*. Simple unmarked deletion of the *HP1284* deletion allele (Δ*HP1284*) in G27 was achieved using primer set HP1284 (**Table C in S1 Text**) as previously described [49]. The SOE-PCR product was used to transform G27*HP1284*::*RC* to generate *HP1284* deletion mutant G27Δ*HP1284*.

#### The complementation of G27Δ*HP1284*

*HPG27_1236* (corresponds to *HP1284*) was amplified from G27 genomic DNA using primers HPG27_1236F and HPG27_1236R. The PCR product was digested with *Xho*I and cloned into the unique *Xho*I site of pHel2_uP to give pHel2_uP_HP1284. The correct orientation of *HP1284* was confirmed by the successful amplification of a 1.2 kb PCR product using primers HPG27_1236F and pHel2F. The pHel2_uP_HP1284 plasmid was introduced into G27Δ*HP1284* by conjugation in a tri-parental mating format as previously described [50] using *E*. *coli* β2150 transformed with pHel2_uP_HP1284 as the donor strain.

#### The single and double deletion of *HP1284* and *waaL* in strain X47

The construct *XHP1284*::*rpsL-cat* that was made to replace *HP1284* in X47 to obtain strain X47Δ*HP1284*. *XHP1284*::*rpsL-cat* construct was made using primer set XHP1284 and methodologies previously described [49]. Similarly the construct *XwaaL*::*kan* was made to replace *waaL* gene in X47 to obtain X47Δ*waaL*. The *XwaaL*::*kan* construct was made using primer set XwaaL. pENT-RK was used as template DNA for the *alpha* cassette. The double deletion mutant X47Δ*HP1284_*Δ*waaL* was obtained by natural transformation of strain X47Δ*HP1284* with *XwaaL*::*kan*.

#### The deletion of *HP0579-HP0580* in strain X47

The construct *XHP0579-80*::*rpsL-cat* was made to replace the *HP0579* and *HP0580* open reading frames in X47 with the *rpsL-cat* cassette to generate strain X47Δ*HP0579-HP0580*. The *XHP0579-80*::*rpsL-cat* construct was made using primer set XHP0579.

### LPS microextraction for silver stain and Western blot

LPS microextraction was prepared as previously described [51]. Briefly, bacterial cells (OD_600_ = 3.0) harvested from CBA plates were suspended in 100 μL LPS lysis buffer (2% SDS, 4% β-mercaptoethanol, 0.1% bromophenol blue, 10% glycerol, 1 M Tris-HCl (pH 6.8)). Samples were heated at 100°C for 10 min. Thereafter, 5 μL proteinase K (PK) (20 mg/mL) was added to the cooled samples, and incubated in a 55°C water bath overnight. The obtained LPS samples were run on 15% SDS-PAGE gels or 16% Tricine-SDS-PAGE gels and visualized by silver stain [52] and by Western blot, using mouse anti-Le^x^ (1:1500) and anti-Le^y^ (1:1500) antibodies (Santa Cruz). After incubation with a secondary rabbit anti-mouse peroxidase-conjugated IgM antibody (1:10,000) (Jackson ImmunoResearch), detection of the HRP conjugates was accomplished by chemiluminescence (Sigma) using a LAS-3000 Intelligent DarkBox (Fujifilm) (software Image reader LAS 3000 V2.2).

### LPS large-scale extraction for structural analysis

LPS from wild-type G27 and X47, G27Δ*HP1284* and X47Δ*HP1284* was extracted using the hot phenol-water method [51] with modifications. The bacterial cells grown on 25 CBA plates (24 h growth) were harvested into 15 mL of 10 mM Tris-HCl buffer (pH 8.0) containing 2% SDS, 4% β-Mercaptoethanol and 2 mM MgCl_2_. After thorough suspension, 100 μL PK (20 mg/mL) was added to the cell mixture and incubated in a 55°C water bath overnight. Subsequently, 2 mL cold sodium acetate (3 M) and 20 mL cold absolute ethanol were added in order and the suspension was allowed to precipitate at -20°C overnight. LPS was then centrifuged down at 10,000 rpm for 10 min at 4°C, the supernatant discarded and the precipitation procedure repeated to remove residual SDS. After the final centrifugation, the pellet was suspended in 10 mL of 10 mM Tris-HCl buffer (pH 7.4). 100 μL DNase I (20 mg/mL) and 100 μL RNase (20 mg/mL) were added and the solution was incubated in a 37°C water bath for 4 h. The LPS mixture and 10 mL 90% liquid phenol were then transferred to a 68°C water bath for 10 min. The preheated phenol was then added to the LPS mixture, and incubated at 68°C for 30 min and then put on ice box for 10 min. The cooled mixture was centrifuged at 4,000 rpm for 60 min at 4°C and the resulting aqueous layer was carefully transferred to a 50 mL Falcon tube. The hot phenol-water extraction was repeated again and the two aqueous layers were combined. Phenol was removed by dialysis against water for 2 days. The LPS sample was then lyophilized and suspended in 10 mL water, and ultracentrifuged at 100,000 *g* for 12 h at 4°C. The resulted gel-like pellet was washed three times in 2 mL chloroform-methanol mixture (1:2 v/v) to remove phospholipids. After air drying, the LPS was resuspended in 3 mL H_2_O and lyophilized.

LPS from G27Δ*waaL* was extracted using the EDTA-promoted method [53] with modifications. The bacterial cells grown on 25 CBA plates (24 h growth) were harvested into 15 mL of 10 mM Tris-HCl buffer (pH 8.0) containing 2mM MgCl_2._ The bacterial cells were sonicated, and DNase I and RNase A were added to a final concentration of 100 μg/mL and 25 μg/mL, respectively. The suspension was incubated in a 37°C water bath for 4 h. After the incubation, 5 mL of 0.5 M EDTA, 2.5 mL of 20% SDS, and 2.5 mL of 10 mM Tris-HCl buffer (pH 8.0) were added to give a final volume of 25 mL, and the pH was raised to 9.5. The solution was vortexed, and centrifuged at 50,000 *g* for 30 min at 20°C to remove peptidoglycan. The supernatant was transferred to a new 50 mL tube, and PK was added to give a final concentration of 200 μg/mL. The sample was then incubated in a 55°C water bath overnight. Two volumes of 0.375 M MgCl_2_ in 95% ethanol were added, and the mixture was cooled in a -20°C freezer for 1 h. After the cooling step, the sample was centrifuged at 12,000 *g* for 15 min at 4°C. The obtained pellet was resuspended in 25 mL of 10 mM Tris-HCl (pH 8.0) containing 2% SDS and 0.1 M EDTA. The sample was then sonicated, and the pH was lowered to 7.0 by the addition of 4 M HCl. The solution was then incubated at 85°C for 30 min to ensure denaturation of SDS-resistant proteins. After cooling, the pH was raised to 9.5 by the addition of 4 M NaOH. PK was then added to give a final concentration of 25 μg/mL, and the sample incubated in a 55°C water bath for 4 h. After the incubation, two volumes of 0.375 M MgCl_2_ in 95% ethanol were added, mixed, and cooled in -20°C freezer for 1 h. The sample was centrifuged at 12,000 *g* for 15 min at 4°C. The obtained LPS pellet was resuspended in 10 mL of 10 mM Tris-HCl (pH 8.0) containing 25 mM MgCl_2_, and subjected to sonication. The LPS solution was ultracentrifuged at 100,000 *g* for 12 h at 15°C. The obtained pellet was washed three times in 2 mL chloroform-methanol mixture (1:2, v/v) to remove phospholipids. After air drying, the LPS pellet was resuspended in 10 mL of 10 mM Tris-HCl (pH 8.0) containing 25 mM MgCl_2_, and ultracentrifugation was repeated as described above. The obtained LPS pellet was resuspended in H_2_O (3 mL) and lyophilized.

### GC-EI-MS trimethylsilyl analysis

Trimethylsilyl derivatised monosaccharides were prepared as previously described [54] and analysed on a PerkinElmer Clarus 500 instrument fitted with a RTX-5-fused silica capillary column. The oven temperature was initially 65°C, and increased to 140°C at the rate of 25°C/min, and then to 200°C at the rate of 5°C/min. The temperature was finally raised to 300°C at a rate of 10°C/min and held for 5 min.

### GC-EI-MS linkage analysis

Partially methylated alditol acetates were prepared as previously described [55]. A Bruker 456-GC/SCION SQ instrument fitted with a Bruker BR-5ms column was used to carry out the experiments. The sample was injected into the column at 60°C and held for 1 min, and the temperature was increased to 300°C over 30 min at a rate of 8°C/min and held for 5 min.

### Methanolysis and mild HF hydrolysis

LPS samples were dissolved in 0.5 M methanolic-HCl solution and incubated at 50°C for 40 min. The supernatant was collected and dried under a stream of nitrogen. The dried methanolysis products were permethylated and analysed by MS. For HF hydrolysis, LPS samples (200 μg) were hydrolysed as previously described [56] except that samples were dissolved in 50 μL of 48% HF and incubated at 4°C for 24 h with reagents being removed under a stream of nitrogen.

### Mild periodate oxidation

The LPS sample (200 μg) was dissolved in 100 μL sodium periodate solution (20 mM, in 100 mM ammonium acetate buffer, pH = 6.5). The solution was incubated at 4°C for 20 h in the dark. The reaction was terminated by adding ethylene glycol and was kept at room temperature for 1 h before lyophilisation. Sodium borohydride (400 μL, 10 mg/mL in 2 M ammonium hydroxide) was added to the sample and incubated at room temperature for 2 h. The reaction was quenched by adding 3–5 drops of acetic acid and purified by Dowex resin.

### Permethylation

Sodium hydroxide (three to five pellets per sample) was crushed in dimethyl sulfoxide (3 mL). The resulting slurry (0.75 mL) and iodomethane (0.85 mL) were added to the sample. After agitating at room temperature (60 min), the reaction was quenched by adding ultrapure water (2 mL) with shaking. The glycans and lipo-glycans were extracted with chloroform (2 mL), and the solution was washed with ultrapure water (2x). The chloroform was then removed under a stream of nitrogen.

### Mass spectrometry

MALDI-TOF spectra were recorded by either a Voyager DE-STRTM MALDI–TOF or a 4800 MALDI-TOF/TOF mass spectrometer (Applied Biosystems, Darmstadt, Germany) with MALDI-TOF/TOF spectra acquired with the latter instrument. The 4700 Calibration standard kit (Applied Biosystems) was used for calibrating the MS mode and fibrinopeptide B (Sigma) was used for calibrating the MS/MS mode. The collision energy for MS/MS was set to 1 kV, and the collision gas was argon. 2, 5-Dihydroxybenzoic acid and 3,4-diaminobenzophenone were used as matrix. Permethylated samples were dissolved in methanol (10 μL) and the solution premixed with matrix (20 mg/mL) with a ratio of 1:1 (v/v) with the mixture (1 μL) being spotted on the plate.

### Micelle NMR

The LPS samples were incorporated into micelles as previously described [57]. Briefly, 6.5 mg LPS sample was mixed with D_38_-DPC (Cambridge Isotope Laboratories. Inc.) at an estimated molar ratio of 1:40 dissolved in deuterated potassium phosphate (50 mM, pD = 6), and transferred to a 5 mm NMR tube. 1- and 2D TOCSY and NOESY NMR spectra were recorded at 30°C using a Bruker Avance III 600MHz NMR spectrometer equipped with a TXI/TCI cryoprobe.

### Determination of polymyxin B MIC

Minimum inhibitory concentrations were determined using polymyxin B Etest strips (Biomérieux). Bacteria from 24 h CBA plate cultures were collected in PBS and standardized to an OD_600_ = 2.0. New blood agar plates were uniformly inoculated with standardized bacterial suspensions using a cotton swab and allowed to dry completely, followed by addition of the Etest strip to the centre of the plate. The plates were incubated at 37°C in a microaerobic atmosphere for 72 h before reading. Each experiment was repeated in triplicate, and the averages and standard deviations are reported.

### Mouse colonisation assays

C57BL/6J female mice aged 6–8 weeks were purchased from Animal Resource Centre, Australia. Each mouse was orally challenged with 0.2 mL bacterial inoculum in HI broth containing 1× of 10^9^ colony-forming units (CFU) of *H*. *pylori* strains harvested from CBA plates after 24 h growth at 37°C under microaerobic conditions. Three independent sets of experiments were performed. In Experiment 1, two groups of mice (n = 5 per group) challenged with wild-type X47 or X47Δ*HP1284* were sacrificed at 2 weeks. In Experiment 2, two groups of mice (n = 10 per group) challenged with wild-type X47 or X47Δ*HP1284* were also sacrificed at 2 weeks. In Experiment 3, four groups of mice (n = 5 per group) challenged with wild-type X47 or X47Δ*waaL* and were sacrificed at 2 and 8 weeks.

After sacrifice, the stomachs were removed from the mice and were opened along the greater curvature. The non-mucosal, squamous forestomach was discarded and the stomach content was gently removed with a sterile loop. Stomach tissue was cut into small pieces and homogenized in 1 mL HI broth by a Tissue lyser (Qiagen). Aliquots (100 μL) of neat, 1× 10^−1^ and 1× 10^−2^ dilutions of the stomach homogenate were spread onto *H*. *pylori* selective plates (CBA containing 5% horse blood, Dent supplement (Oxoid), nalidixic acid at 10 μg/mL and bacitracin at 100 μg/mL. After culture for 4–5 days, the number of colonies was counted and CFU/stomach was calculated to determine bacterial load.

### Ethics statement

Mouse experimental procedures were reviewed and approved by the Institutional Animal Care and the Animal Ethics Committee of the University of Western Australia (AEC Approval No: RA 03/100/1085) and adhered to the Australian code for the care and use of animals for scientific purposes (8^th^ Edition, 2013) and the Animal Welfare Act (2002) of Western Australia. Animals were euthanased using Isofluorane inhalation and cervical dislocation.

## Supporting information

S1 Text (DOCX)
