# Supplementary material for: The redefinition of Helicobacter pylori lipopolysaccharide O-antigen and core-oligosaccharide domains
Source: PLoS Pathog. 2017 Mar 17;13(3):e1006280. doi: 10.1371/journal.ppat.1006280 (PMC5371381; doi:10.1371/journal.ppat.1006280)
Supplement: S1 Text — (DOCX) [file ppat.1006280.s001.docx]

**S1 Text**

**Figure legends**:

**Figure A**. GC-EI-MS TMS analysis of the monosaccharides from *H. pylori* G27 wild-type and LPS mutants. Full standards (A); G27 wild-type (B); G27∆*HP1284* (C); and G27∆*waaL* (D). The identities and retention of the monosaccharides are annotated on the chromatograms. Note that multiple peaks can correspond to each monosaccharides.

**Figure B**. LPS profiles of 26695 and G27 wild-type. LPS preparations from *H. pylori* wild-type strains 26695 (A) and G27 (B) were analyzed by silver stain and Western blot using anti-Lex and anti-Ley antibodies.

**Figure C**. MS analysis of G27∆*HP1284* LPS. The MALDI-TOF spectra of HF hydrolysed and periodate oxidised are shown in (A) and (B), respectively. The MS peaks corresponding to sodiated glycans are coloured red, and annotated with m/z values and glycan structures. Both data are similar of LPS extracted from the WT G27. The MALDI-TOF/TOF spectrum of the MS peak at *m/z* 1188.6 (the sodiated version of the peak at *m/z* 1880.6 in main text **Figure 4A**) is shown in (C). The fragmentation pattern strongly suggests a sequence of GlcNAc-Hep-Hep-KDO.

**Figure D**. LPS profiles of X47 wild-type and LPS mutant strains. LPS preparations from wild-type and mutant strains were analyzed by high resolution Tricine-SDS-PAGE. Lane 1, wild-type; Lane 2, *HP1284* deletion; Lane 3-4 and 7, *waaL* deletion; Lane 5-6 and 8, *HP1284* and *waaL* double deletion.

**Figure E**. MS analysis of X47 wild-type and X47∆*HP1284* LPS. The MALDI-TOF spectra of WT X47 and X47∆*HP1284* LPS are shown in (A) and (B), respectively. The LPS samples were methanolysed and permethylated before MS analysis. Red MS peaks correspond to sodiated glycans, and they are annotated with *m/z* values and glycan structures. These data strongly indicate that the X47 samples share very similar architectures with corresponding G27 strains.


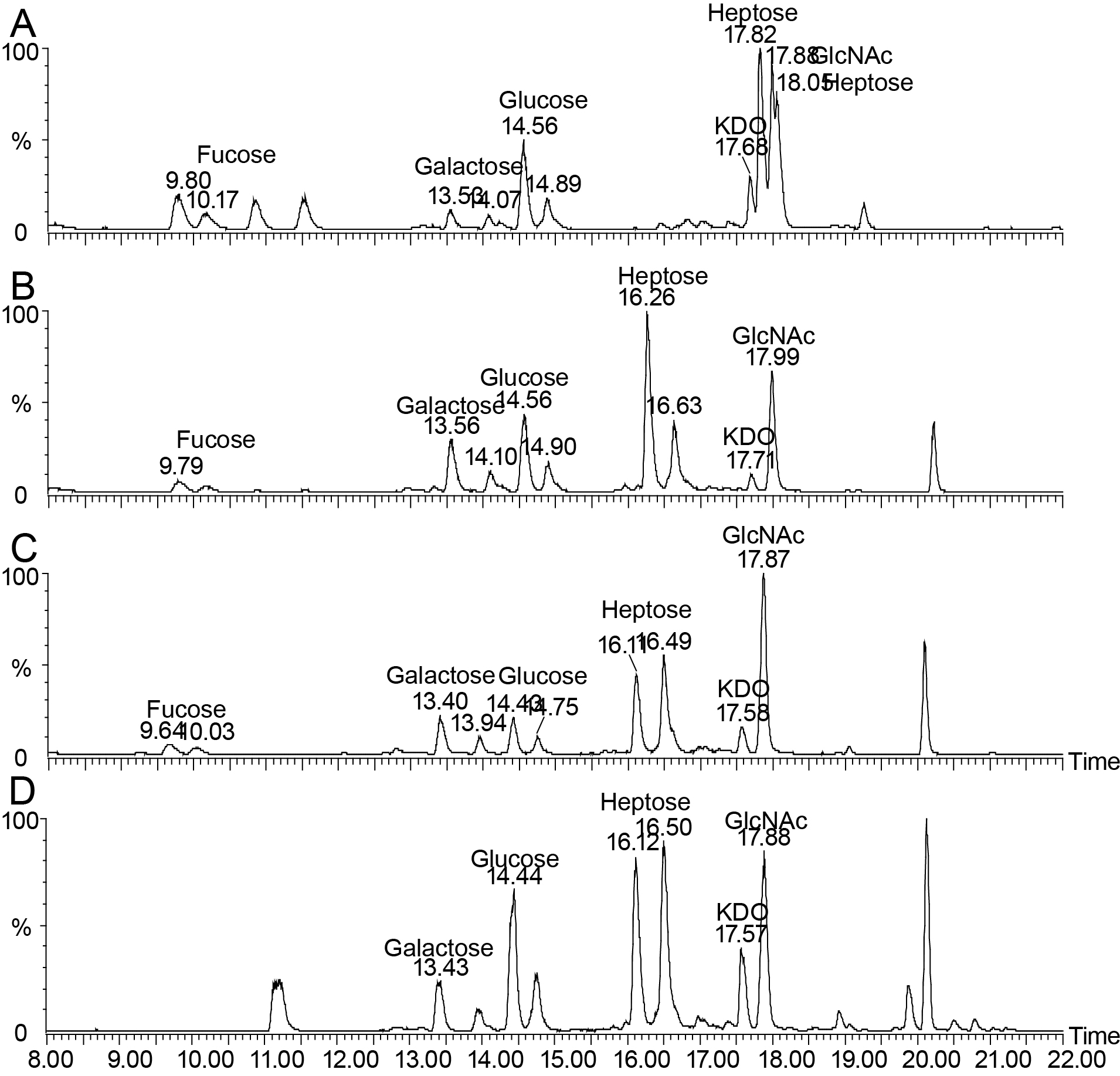


**Figure A**.


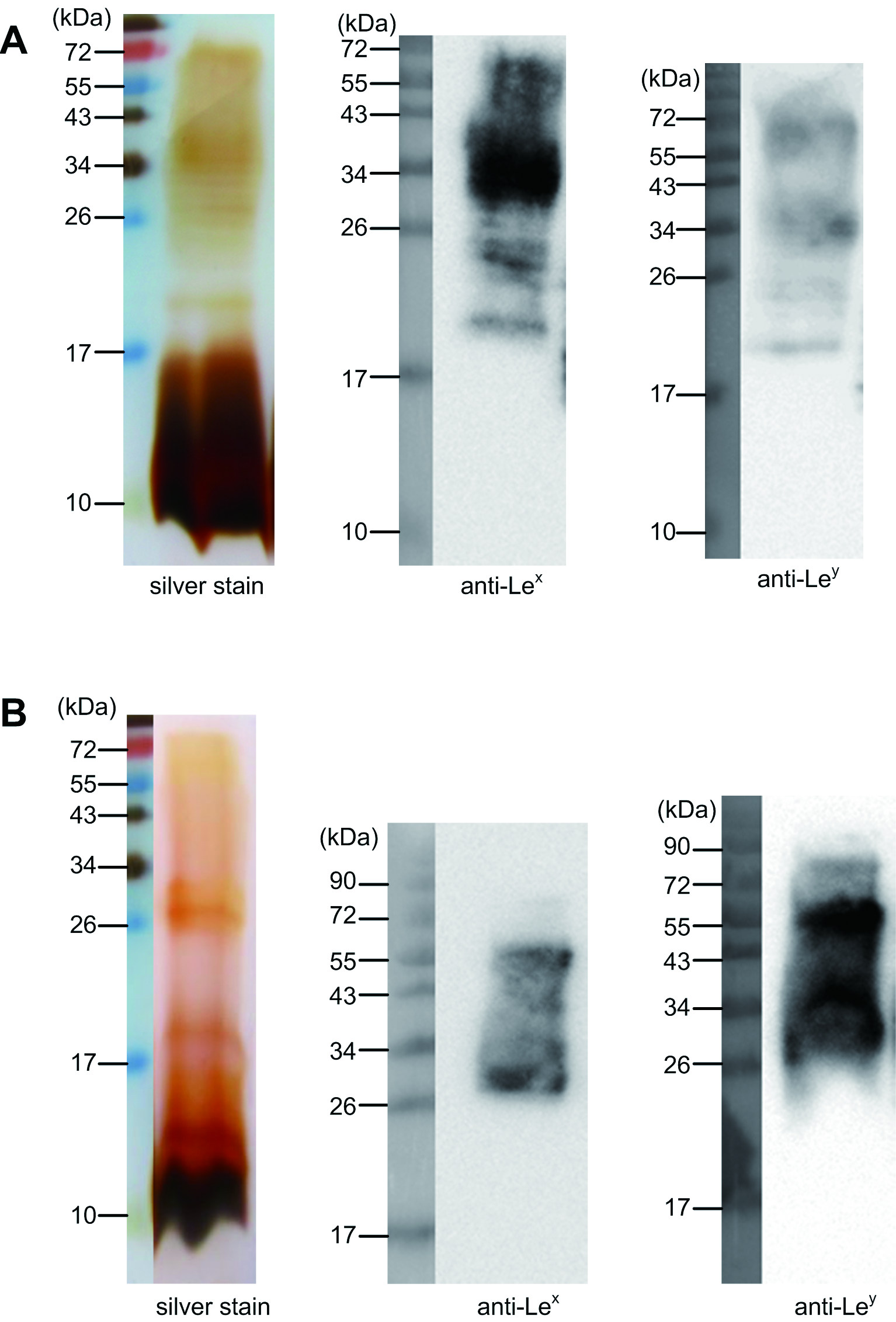


**Figure B**.


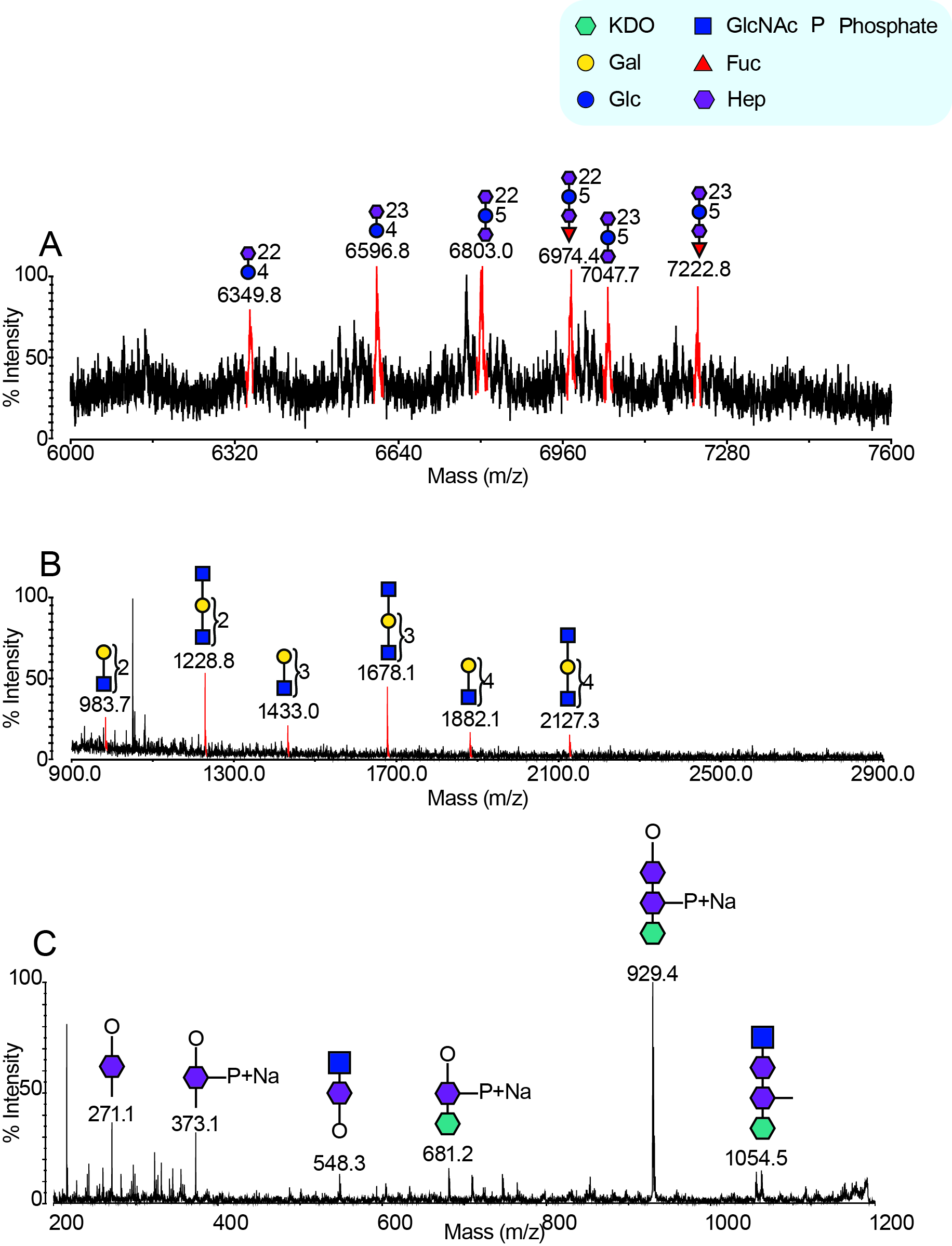


**Figure C**.


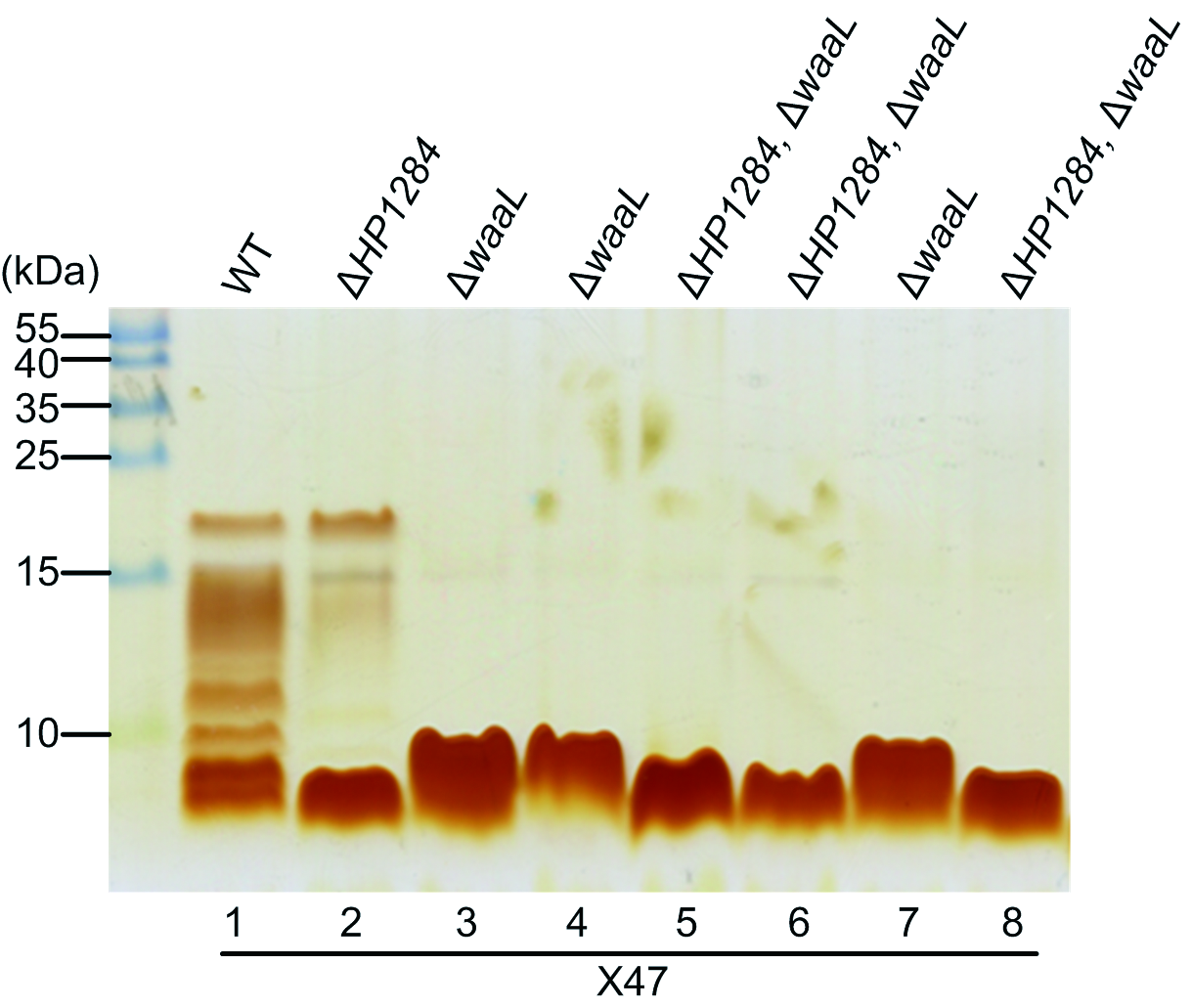


**Figure D**.


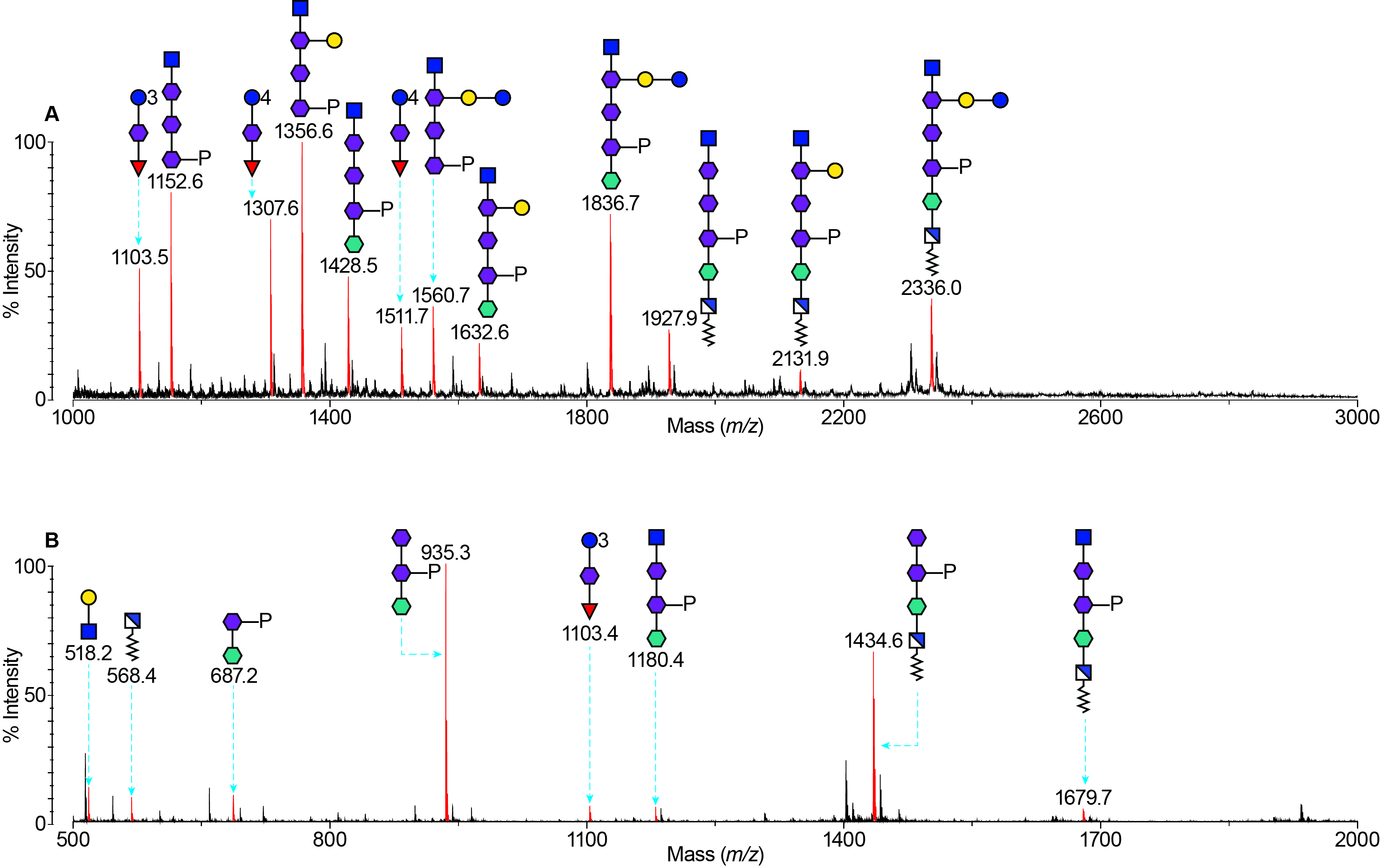


**Figure E**.

**Table A**. Linkage analysis of LPS samples

| Monosaccharides | RT (min) | Characteristic Ions |
| --- | --- | --- |
| t-Fuc | 12.8 | 89+102+115+118+131+175 |
| 3-Fuc | 14.0 | 87+118+131+174+190+234+247 |
| t-Glc | 14.2 | 87+102+118+129+145+161+162+205 |
| t-Gal | 14.5 | 87+102+118+129+145+161+162+205 |
| 3-Glc | 15.4 | 101+118+129+161+202+217+234 |
| 4-Gal | 15.4 | 87+99+102+113+118+129+131+162+173+233 |
| 2-Gal | 15.6 | 87+88+101+129+130+161+190+205+234 |
| 3-Gal | 15.7 | 101+118+129+161+202+217+234 |
| 6-Glc | 15.7 | 87+102+118+129+162+189+233 |
| 2-DD-Hep | 17.0 | 89+101+130+190+205+234+249+306+350 |
| 3-DD-Hep | 17.2 | 101+118+202+205+234+276+290+318+350 |
| 2-Hep | 17.3 | 89+101+130+190+205+234+249+306+350 |
| 7-Hep | 17.5 | 102+117+118+162+189+233+277+321+322 |
| 3-Hep | 17.6 | 101+118+202+205+234+276+290+318+350 |
| t-GlcNAc | 17.8 | 117+129+143+145+159+203+205+247+258 |
| 2,7-Hep | 18.4 | 117+130+190+201+233+277+350 |
| 4-GlcNAc | 18.6 | 117+129+143+159+203+233 |
| 3-GlcNAc | 19.1 | 75+117+129+159+181+197+203+231+244+258+272+275+318 |
| 3,4-GlcNAc | 19.4 | 75+117+142+159+189+346 |

**Table B**. ^1^H Chemical shifts of G27*∆waaL* LPS

| Residue | H1 | H2 | H3 | H4 |
| --- | --- | --- | --- | --- |
| 6-linked β GlcN (major) | 4.43 | 3.98 | 5.28 | 4.20 |
| 6-linked β GlcN (minor) | 4.42 | 4.01 | 5.32 | 4.16 |
| 6-linked α GlcN 1-P | 5.48 | 3.81 | 3.63* | 3.54* |
| 5-linked KDO | − | − | 2.10 (eq.);1.86(ax.) | 4.16 |
| 7-linked α-D,D-Hep | 5.04 | 4.09 | ND | ND |
| 2-linked α-L,D-Hep | 5.43 | 4.09 | 3.99 | 3.82 |
| 3-linked α-L,D-Hep | 5.08 | 4.16 | ND | ND |
| 4-linked β Gal | 4.50 | 3.60 | 3.74 | 4.02 |
| terminal α Glc | 4.93 | 3.52 | 3.75 | 3.45 |

* These assignments may be reversed

ND = not determined

**Table C**. Plasmids and bacterial strains used in this study

| Plasmid or strain name | Description | Source or reference |
| --- | --- | --- |
| Plasmid |  |  |
| pENT-RC | pEntranceposon, containing *rpsL-cat* flanked by MuA transposase recognition site | Ondek |
| pENT-RK | pEntranceposon, containing *rpsL-alpha* flanked by MuA transposase recognition site | Ondek |
| pDifWT-RC | *rpsL-cat* cassette flanked by *H. pylori* *difH* sequence | [[1](#_ENREF_1)] |
| pWaaL-AB | pGEM^®^-T Easy vector containing sequences flanking *HPG27_389*, separated by a *Bam*HI restriction site | This work |
| pWaaL-AB-difH-RC | Derivative of pWaaL-AB, with *difH-rpsL-cat-difH* inserted between *HPG27_389* flanking sequences at the *Bam*HI site | This work |
| p0579-AB | pGEM^®^-T Easy vector containing sequences flanking *HPG27_538*, separated by a *Bam*HI restriction site | This work |
| p0579-AB-difH-RC | Derivative of p0579-AB, with *difH-rpsL-cat-difH* inserted between *HPG27_538* flanking sequences at the *Bam*HI site | This work |
| pCR2.1-1284 | pCR^®^2.1-TOPO^®^ vector containing sequence *HP1284* | This work |
| pCR2.1-1284-RC | pCR2.1-1284 containing the *rpsL-cat* insertion within the sequence *HP1284* | This work |
| pHel2 | *E. coli-H. pylori* shuttle plasmid | [[2](#_ENREF_2)] |
| pHel2_uP | Derivative of pHel2 containing core urease promotor | This work |
| pHel2_uP_HP1284 | pHel2_uP expressing *HP1284* | This work |
| *H. pylori* strain |  |  |
| G27^Str^ | Streptomycin resistant | [[3](#_ENREF_3)] |
| G27Δ*waaL* | Clean deletion of *waaL* | This work |
| G27Δ*HP0826* | Clean deletion of *HP0826* | This work |
| G27Δ*HP1191* | Clean deletion of *HP1191* | This work |
| G27*HP1284::RC* | Insertion mutation of *HP1284* by *rpsL-cat* | This work |
| 26695^Str^ | Streptomycin resistant | [[4](#_ENREF_4)] |
| 26695*HP1284::RC* | Insertion mutation of *HP1284* by *rpsL-cat* | This work |
| G27Δ*HP1284* | Clean deletion of *HP1284* | This work |
| G27Δ*HP1284* complementation | The conjugation of pHel2_uP_HP1284 into G27Δ*HP1284* | This work |
| X47 | Naturally streptomycin resistant | [[5](#_ENREF_5),[6](#_ENREF_6)] |
| X47Δ*HP1284* | Replacement of *HP1284* with *rpsL-cat* | This work |
| X47Δ*waaL* | Replacement of *waaL* with *alpha* | This work |
| X47Δ*HP1284_*Δ*waaL* | Double deletion of *HP1284* and *waaL* | This work |
| *E. coli* strain |  |  |
| β2150 donor strain | Δ*dapA*, erm^r^ | [[2](#_ENREF_2)] |
| β2150 helper strain | Δ*dapA*, kan^r^, erm^r^ | [[2](#_ENREF_2)] |

**Table D**. Oligonucleotides used in this study

| **Primer set** | **Sequence (5'--> 3')** | **Function of PCR product** |
| --- | --- | --- |
| **WaaL** |  | Construction of pWaaL-AB-difH-RC |
| WaaL-F | CCTAGTAGAGCCATTAGTAACCAC |  |
| WaaL-BamHI-F | GTTTGGATCCCATCAAAGAGTTTGATGTTAGTC |  |
| WaaL-BamHI-R | CAAACTCTTTGATGGGATCCAAACTCGCCACACCCTAAAACC |  |
| WaaL-R | GAGTGTAGCGAGAATCAGTG |  |
| **HP0579** |  | Construction of p0579-AB-difH-RC |
| HP0579-F | CACGCAACTCACTATCCCTAAACC |  |
| HP0579-BamHI F | CAAGTGATTAATGGGATCCCGCTTATGAAATCCCTATCCTATG |  |
| HP0579-BamHI R | GGATTTCATAAGCGGGATCCCATTAATCACTTGAGAAGGGATTTTAAATAAGC |  |
| HP0579-R | GCTCGTTAGCCCTATGGAATC |  |
| HP1284 F | CGCTAAAAACAGCCAAGAACAAC | Construction of pCR2.1-1284 |
| HP1284 R | GTAACGACTATTAAAGAACCTTC |  |
| **HP1284** |  |  |
| HP1284 clean KO F1 | GCAAACGCCAAACTAACACC | SOE PCR for clean deletion of *HP1284* |
| HP1284 clean KO F2 | GACAAAAAGCCAGCCGTCATCTTG |  |
| HP1284 clean KO F3 | CTTAGGCGGAGTTATTTGATAAGAGACTTTCTACTATAATCAC |  |
| HP1284 clean KO R1 | GAAAGGCGTGAGCCATTAACG |  |
| HP1284 clean KO R2 | GCTATACCCTTGATAGTAGC |  |
| HP1284 clean KO R3 | GTCTCTTATCAAATAACTCCGCCTAAGAGATCAAATTTTTTGTC |  |
| HPG27_1236F | GACGACCTCGAGATGGATTTTGTAGGGTTTGAAG | Construction of pHel2_uP_HP1284 |
| HPG27_1236R | GTCGTCCTCGAGCTACATCTTGCGGTAATTG |  |
| pHel2F | CAGAAATTCGGATCTTCCATAC | Check the correct orientation of *HP1284* in pHel2_uP_HP1284 |
| **XHP1284** |  | Construction of *XHP1284::rpsL-cat* |
| XHP1284-1 | ACGGTATCGATGGTTTTGCAAACACCAAACTAAC |  |
| XHP1284-2 | GTGTTTAATCCATAGTTATAAAGCATCAACCCCGCCTAAGAGATC |  |
| XHP1284-3 | CAAGACTTGCTGAATAAATAAAATCCGACAACAAGTATTACACAAACAAC |  |
| XHP1284-4 | CTCCACCGCGGCAACTAACGCTAACTTCTCTAC |  |
| XHP1284-7 | GGTAAAATACTCCTAATCATCTG |  |
| XHP1284-8 | CTGTAATTCTTGAGCTATCTCTG |  |
| rcat-5 | GATGCTTTATAACTATGGATTAAACAC |  |
| rcat-6 | GGATTTTATTTATTCAGCAAGTCTTG |  |
| **XwaaL** |  | Construction of *XwaaL::kan* |
| XwaaL-1 | TGCGTTAAAATCATTCTGGTGGTG |  |
| XwaaL-2 | GGGTACCGAGCTCGAATTCTTTCAACACAAACTCGCCATACC |  |
| XwaaL-3 | GGAATAATGACCCGGGGATCCCATTTTTGGCATCGGGTTTGAC |  |
| XwaaL-4 | TCATTGAGCGTGAAATGCGATTC |  |
| XwaaL-7 | ATAGCGCGATAGAAATCGTGAG |  |
| XwaaL-8 | CCATTAAAGCCATGTTGTAACCAAG |  |
| kan-5 | GAATTCGAGCTCGGTACCC |  |
| kan-6 | GGATCCCCGGGTCATTATTCC |  |
| **XHP0579** |  | Construction of *XHP0579-80::rpsL-cat* |
| XHP0579-1 | TCTCAGTAAGCCCTTGATTGAC |  |
| XHP0579-2 | GTGTTTAATCCATAGTTATAAAGCATCAGGTTCCAAGATAGCGTTTC |  |
| XHP0579-3 | CAAGACTTGCTGAATAAATAAAATCCGTCTTTTTTAAGACTTTATCTCTTG |  |
| XHP0579-4 | AATCTTGTAAAGCGCTCGTTAG |  |

**Supplementary References**

1. Debowski AW, Carnoy C, Verbrugghe P, Nilsson HO, Gauntlett JC, et al. (2012) Xer recombinase and genome integrity in *Helicobacter pylori*, a pathogen without topoisomerase IV. PLoS One 7: e33310.

2. Heuermann D, Haas R (1998) A stable shuttle vector system for efficient genetic complementation of *Helicobacter pylori* strains by transformation and conjugation. Mol Gen Genet 257: 519-528.

3. Baltrus DA, Amieva MR, Covacci A, Lowe TM, Merrell DS, et al. (2009) The complete genome sequence of *Helicobacter pylori* strain G27. J Bacteriol 191: 447-448.

4. Dailidiene D, Dailide G, Kersulyte D, Berg DE (2006) Contraselectable streptomycin susceptibility determinant for genetic manipulation and analysis of *Helicobacter pylori*. Appl Environ Microbiol 72: 5908-5914.

5. Handt LK, Fox JG, Stalis IH, Rufo R, Lee G, et al. (1995) Characterization of feline *Helicobacter pylori* strains and associated gastritis in a colony of domestic cats. J Clin Microbiol 33: 2280-2289.

6. Kleanthous H, Tibbitts TJ, Gray HL, Myers GA, Lee CK, et al. (2001) Sterilizing immunity against experimental *Helicobacter pylori* infection is challenge-strain dependent. Vaccine 19: 4883-4895.
